# Comparison of High-Protein, Intermittent Fasting Low-Calorie Diet and Heart Healthy Diet for Vascular Health of the Obese

**DOI:** 10.3389/fphys.2016.00350

**Published:** 2016-08-29

**Authors:** Li Zuo, Feng He, Grant M. Tinsley, Benjamin K. Pannell, Emery Ward, Paul J. Arciero

**Affiliations:** ^1^Radiologic Sciences and Respiratory Therapy Division, School of Health and Rehabilitation Sciences, The Ohio State University College of Medicine, The Ohio State University Wexner Medical CenterColumbus, OH, USA; ^2^Department of Kinesiology, California State University, ChicoChico, CA, USA; ^3^Human Nutrition and Metabolism Laboratory, Health and Exercise Sciences Department, Skidmore CollegeSaratoga Springs, NY, USA; ^4^Department of Kinesiology and Sport Management, Texas Tech UniversityLubbock, TX, USA

**Keywords:** arterial compliance, cholesterol, lipids, weight loss, weight relapse

## Abstract

**Aim:** It has been debated whether different diets are more or less effective in long-term weight loss success and cardiovascular disease prevention among men and women. To further explore these questions, the present study evaluated the combined effects of a high-protein, intermittent fasting, low-calorie diet plan compared with a heart healthy diet plan during weight loss, and weight loss maintenance on blood lipids and vascular compliance of obese individuals.

**Methods:** The experiment involved 40 obese adults (men, *n* = 21; women, *n* = 19) and was divided into two phases: (a) 12-week high-protein, intermittent fasting, low-calorie weight loss diet comparing men and women (Phase 1) and (b) a 1-year weight maintenance phase comparing high-protein, intermittent fasting with a heart healthy diet (Phase 2). Body weight, body mass index (BMI), blood lipids, and arterial compliance outcomes were assessed at weeks 1 (baseline control), 12 (weight loss), and 64 (12 + 52 week; weight loss maintenance).

**Results:** At the end of weight loss intervention, concomitant reductions in body weight, BMI and blood lipids were observed, as well as enhanced arterial compliance. No sex-specific differences in responses were observed. During phase 2, the high-protein, intermittent fasting group demonstrated a trend for less regain in BMI, low-density lipoprotein (LDL), and aortic pulse wave velocity than the heart healthy group.

**Conclusion:** Our results suggest that a high-protein, intermittent fasting and low-calorie diet is associated with similar reductions in BMI and blood lipids in obese men and women. This diet also demonstrated an advantage in minimizing weight regain as well as enhancing arterial compliance as compared to a heart healthy diet after 1 year.

## Introduction

In the United States, the prevalence of overweight and obesity is over 60% in adults (Flegal et al., 2010), which subsequently contributes to metabolic and cardiovascular diseases (CVD). Obesity is often understood to coexist with numerous cardiovascular risk factors (Wilson et al., 2002), and is associated with multiple inflammatory markers and cytokines that potentially contribute to the adverse cardiovascular outcomes in individuals with obesity (Van Gaal et al., 2006). Men suffer from a higher prevalence of these disorders than women, indicating that sex-based differences may play a role in cardiometabolic health (Lönnqvist et al., 1997; Blaak, 2001). Many short-term weight loss (WL) interventions are effective at improving cardiovascular/metabolic disease risk factors [e.g., body fat, total cholesterol (TC), and triglycerides (TG; Kelishadi et al., 2008; Clifton et al., 2009; Klempel et al., 2012; Mozaffarian et al., 2015; Pedersen et al., 2015)]. One popular form of WL diet is intermittent fasting (IF), which utilizes repeated short-term fasts to reduce energy intake, promote WL, and improve the lipid profile (Tinsley and La Bounty, 2015). IF has been shown to be as effective as continuous energy restriction in terms of WL, reducing TG, low-density lipoprotein (LDL) cholesterol, and blood pressure (BP; Harvie et al., 2011; Varady, 2011). However, the impact of IF on vascular compliance, an indicator of cardiovascular disease risk (Vlachopoulos et al., 2010), has not been examined. There is also limited information concerning whether men and women differ in their responses to short-term IF. Additionally, IF diets have not been examined in combination with high protein intake aimed at promoting cardiovascular health. Thus, the first aim of this study is to examine if there are sex differences in terms of lipid profile, and arterial compliance following a short-term high-protein, intermittent fasting, low-calorie (HP-IF-LC) diet.

WL induced by nutritional interventions, such as a HP-IF and a traditional heart healthy (HH) diet, has demonstrated beneficial effects on minimizing cardiovascular risk factors (Arciero et al., 2006; Camhi et al., 2010; Song et al., 2016). HH diets exert a protective effect on the heart, as evidenced by reduced rates of myocardial infarction (Hansen-Krone et al., 2012). On the other hand, HP diets contribute to WL and weight loss maintenance (WL-M) by increasing the metabolic rate (Leidy et al., 2015). HP diets also induce extra energy expenditure via protein and urea synthesis as well as gluconeogenesis (Westerterp-Plantenga et al., 2009). Although multiple studies have demonstrated the short-term benefits of HP and IF diets, there is little research focusing on the combined or long-term effects of HP-IF diet on blood lipids, arterial compliance and CVD risk reduction in adults with obesity during and after a short-term WL intervention (Mattson and Wan, 2005; Varady, 2011). Clinical trials have demonstrated the independent effects of either HP, IF, or LC diets on improving cardiovascular outcomes in individuals with obesity (Katare et al., 2009; Harvie et al., 2011; Damsgaard et al., 2013; de Luis et al., 2016). However, few studies emphasize the combined effects of each of these dietary strategies on long-term WL-M and cardiovascular health. Higher protein intake during IF with low energy intake may be beneficial for WL and WL-M due to improved preservation of fat-free mass and resting metabolic rate. Therefore, the second major aim of the current study was to compare the effects of two different WL-M strategies (HP-IF vs. HH) on blood lipids, and arterial compliance in adults with obesity following an initial short-term HP-IF-LC WL diet.

## Materials and methods

### Participants

Eligible volunteers were randomly recruited from the Saratoga Springs, NY area. Participants were eligible for inclusion in the study if they were healthy non-smokers, but were overweight or obese. A comprehensive medical examination/history assessment was performed by their physicians. Individuals were excluded from participation in the study if they had any previous cardiovascular or metabolic disease, or were receiving hormone therapy which could influence weight status, central adiposity and CVD risk factors measured in this study. Additionally, only individuals who were either sedentary or lightly active (<30 min, 2 days/week of organized physical activity), weight stable (± 2 kg during the past 6 months), middle-aged, and non-alcoholic, based on self-report, were eligible for inclusion in the study. Every participant provided written informed consent in accordance with the Skidmore College Human Subjects Review Board prior to participation, and the study was approved by the Human Subjects Institutional Review Board of Skidmore College (IRB #: 1307-347). All experimental procedures were performed in adherence with New York State regulations and the Federal Wide Assurance, which are consistent with the National Commission for the Protection of Human Subjects of Biomedical and Behavioral Research, and in agreement with the Helsinki Declaration (revised in 1983). This trial was registered at clinicaltrials.gov as NCT02525419.

### Experimental design and study timeline

**Figure 1:**
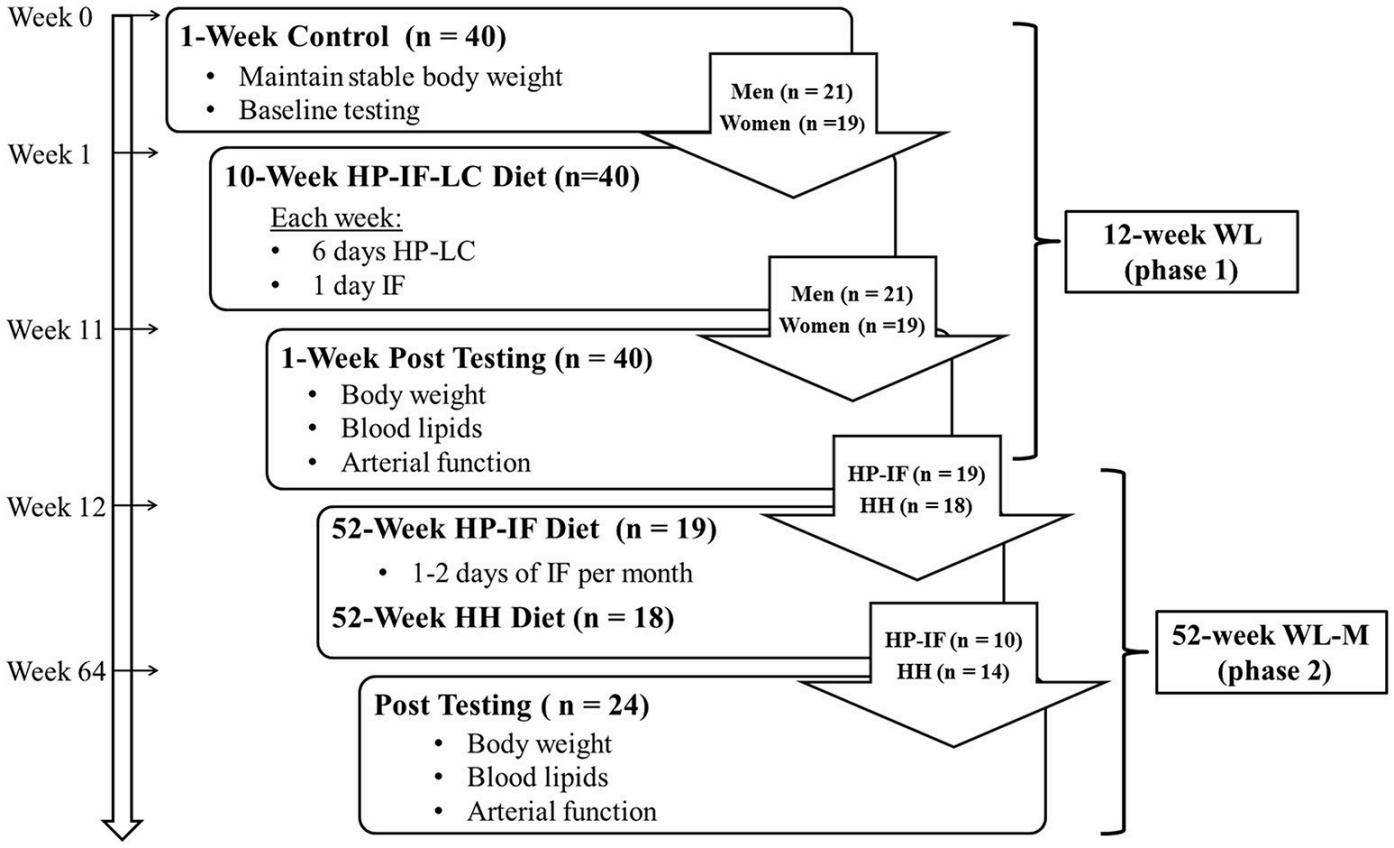
**Schematic illustrating the experiment timeline comprised of a 12-week WL (phase 1), followed by a 52-week WL-M (phase 2)**. HP-IF-LC, high-protein, intermittent fasting, low-calorie; HH diet, heart healthy diet; WL, weight loss; WL-M, weight loss maintenance.

Forty subjects with obesity were enrolled as a single cohort in this 64-week dietary regimen, splitting into two consecutive intervention phases: (a) 12-week WL (Phase 1) with HP-IF-LC diet (1-week baseline control, 10-week WL, 1-week post-testing) comparing men and women, and (b) a 1-year WL-M (Phase 2) comparing two diets (HP-IF vs. HH). All laboratory testing procedures were completed following baseline control (week 0), WL (week 12), and WL-M (week 64), as shown in Figure 1. During baseline control, the height, and body weight were recorded for each participant. In order to maintain stable weight, subjects were asked to maintain routine eating patterns and record dietary food logs for 2 days during baseline control period. In order to verify sedentary/low activity levels, all participants wore an Actical accelerometer (Bio-Lynx Scientific Equipment Inc., Montreal Quebec, Canada) around their waist for 2 days during weeks 0, 5, and 10. At weeks 0, 12, and 64, body weight was assessed between 6:00 and 9:00 a.m., after an overnight fast. Subjects then rested in a supine position for 15-min in a quiet and dimly lit room before a fasted blood draw was performed for assessment of blood lipids and measures of arterial compliance were obtained (see **Laboratory Testing Procedures**). Following control baseline testing, participants were provided with detailed instructions on their WL dietary guidelines (see **Dietary Intervention**) and scheduled their weekly meeting with a licensed registered dietitian. At the beginning of WL-M (Phase 2), all subjects continued to meet with a dietitian, but on a monthly basis.

### Rationale

A HH diet that meets the guidelines of the American Heart Association is comprised of 50–60% of energy from carbohydrates and approximately 15% from protein, with the remaining 25–35% from healthy fats. This diet is often recommended to improve cardiovascular health independent of WL (Fagerberg et al., 1984; Trumbo et al., 2002; Lichtenstein et al., 2006). Thus, it is of great interest to systematically compare the relative effectiveness of this HH diet and other diets, such as a HP-IF diet, in individuals with obesity during WL and long-term WL-M. In this context, using a two-phase study design, our first objective was to determine whether men and women with obesity demonstrate similar cardiovascular health improvements following a 12-week HP-IF-LC diet. Thereafter, we aimed to quantitatively compare the effectiveness of 52 weeks of a HP-IF vs. HH diet on WL-M and maintenance of cardiovascular enhancements produced by WL.

### Dietary intervention

#### WL (Phase 1, weeks 1–12): HP-IF-LC diet

Beginning at week 1, subjects consumed a HP-LC diet 6 days per week, along with an IF diet on the remaining day of the week. This diet was adhered to in conjunction with weekly counseling sessions with a registered dietitian. The composition of the HP-LC diet and the timing of meals are shown in Table 1. In order to induce an energy deficit without compromising lean body mass, the daily macronutrient distribution (30% protein, 45% carbohydrate, and 25% fat) has been used in this phase based on our previous study (Arciero et al., 2014). Total energy intake during HP-LC was 1200 and 1500 kcal per day for females and males, respectively. The eating schedule was designed to produce a regular frequency of meals and protein consumption during the WL and WL-M interventions. During WL, each meal consisted of approximately 20–30 g servings of high-quality protein in either whole food or supplement form. Subjects were told to eat 4–5 meals per day on HP-LC days, as depicted in Table 1. On the one IF day per week, daily energy intake consisted of 330 and 430 kcal for women and men, respectively. The composition of the diet for the IF day is depicted in Table 2.

**Table 1:** **Composition of high-protein low-calorie diet**.

**Meal and recommended time of consumption**	**Energy (kcal)^a^**	**Description**
Breakfast (6:00–8:00 A.M.)	240	Liquid meal replacement^b^
Lunch (11:00 A.M.–1 P.M.)	240	Liquid meal replacement^b^
Afternoon snack^c^ (2:00–4:00 P.M.)	150	Low-glycemic protein wafers or whole-food high protein snack^f^^g^
Dinner^d^ (5:00–7:00 P.M.)	450 or 600	
Evening snack (9:00–10:00 P.M.)	270	Meal replacement bar^e^

a *The total daily macronutrient distribution for the HP-LC diet shown above was 30% protein, 45% carbohydrate, and 25% fat, and total energy intake for males and females was ~1500 and 1200 kcal, respectively. In addition to following this HP-LC diet 6 days per week, an IF diet was followed for one day per week*.

b *Isalean Shake, Isagenix LLC, Chandler, AZ, USA*.

c *Afternoon snack was consumed by males only*.

d *Females consumed 450 kcal meal while males consumed 600 kcal meal*.

e *Isalean Bar, Isagenix LLC, Chandler, AZ, USA*.

f *Snack components for men: 2 hard-boiled egg with 1/3 cup fruit; 6 oz. cottage cheese with 1/3 cup fruit; 1 tbsp of nuts and 1/3 cup of fresh veggies or fruit; 1 slice of whole grain bread with 1.5 tsp nut butter; 1/2 cup Fresh fruit or veggies with 1/3 cup hummus; 8 oz plain greek yogurt; ¼ cup granola*.

g *Snack components for women:1 hard-boiled egg with ¼ cup fruit; 4 oz. cottage cheese; ¼ cup fruit; 4 oz. plain non-fat greek yogurt; ¼cup granola; ½ slice bread; ¾ tsp nut butter; ¼ cup fruit or veggies with 1/5 tbsp hummus*.

**Table 2:** **Composition of intermittent fasting diet**.

**Food item/dietary supplement**	**Consumption frequency**	**Description^a^**
Whole-food high protein snack	1/day	100 or 200 kcal for females and males, respectively
Anti-oxidant rich powder^b^	6/day	120 kcal total
Low-glycemic protein wafers^b^	3/day	90 kcal total
Micronutrient supplement^c^	2/day	Contains vitamins, minerals, phytonutrients, antioxidants, and essential fatty acids
Herbal supplement^d^	1/day	Multiple ingredients, such as wolfberry, kiwi, rhodiola root, harada, tribulus, and maca root

a *During the Phase 1 (weight loss) of the study, participants performed one day of IF per week, which consisted of a total energy intake of 330 kcal/d for women and 430 kcal/d for men*.

b *Isalean Shake, Isagenix LLC, Chandler, AZ, USA*.

c *Ageless Essentials with Product B, AM & PM, Isagenix LLC, Chandler, AZ, USA; consumed on IF and non-IF days*.

d *Ionix Supreme, Isagenix LLC, Chandler, AZ, USA; consumed on IF and non-IF days*.

#### WL-M (Phase 2, weeks 13–64): HP-IF and HH diets

Starting at week 13, participants chose whether to enter the HP-IF or HH diet group for Phase 2 of the study. Both groups (*n* = 10 for HP-IF; *n* = 14 for HH) received monthly dietary counseling from a registered dietitian, and subjects in both groups reported continued desire to lose weight after the initial 12 weeks. In order to resemble free-living conditions (i.e., conditions without excessive supervision), subjects were instructed to follow the guidelines of their respective diets without restrictions on physical activity or total food intake. However, they were encouraged to stay at an intake level necessary for weight maintenance, based on their calculated energy needs [measured resting metabolic rate (RMR) X Activity Factor]. The HP-IF group were provided 2 meal replacements per day (either two protein powder packets or one protein powder packet and one meal replacement bar) while the remaining 2–3 meals were whole foods, as guided by a dietitian. HP-IF subjects also performed IF 1 to 2 days per month.

The HH group followed the dietary guidelines that are in compliance with the National Cholesterol Education Program Therapeutic Lifestyle Changes diet (i.e., < 35% of kcal as fat; 50–60% of kcal as carbohydrates; < 200 mg/dL of dietary cholesterol; and 20–30 g/day of fiber). Both groups (HH and HP-IF) had monthly meetings with a registered dietitian to establish healthy eating choices that were compliant with their meal plans. Additional counseling with the registered dietitian was made available to participants if necessary. During WL-M, the same timing of meals as during WL was implemented (i.e., 4–5 meals per day evenly spaced throughout the day). The only exception was during IF days for the HP-IF subjects.

#### Compliance

To encourage compliance, all subjects had weekly meetings with a registered dietitian during WL and monthly meetings during WL-M to incorporate healthy eating strategies while consuming their appropriate diet. Additionally, all subjects were given detailed written and verbal instructions for each diet plan (HP-IF-LC; HP-IF; HH). For example, participants were informed that HP-IF was designed to maintain intake close to the 1.8 g protein/kg body weight (BW), whereas the HH was designed to deliver 1.0 g protein/kg BW. Furthermore, as stated above, there were no differences between the subjects choosing HH or HP-IF diets for the WL-M phase. Monitoring of the meal plans was performed through daily subject-researcher interaction (e.g., telephone conversations, 2-day food diary analysis, weekly dietary intake journals inspections, distribution of weekly meal/supplement containers, return of empty packets and containers, and monthly group meetings). The PI (PJA) and/or an investigator held weekly meetings with all participants to verify compliance with the dietary meal plans, clarify dietary guidelines, and answer questions. Participants demonstrated a high compliance rate (>90%), which was defined as consuming more than 85% of their respective meals/supplemented feedings. Subjects were considered non-compliant if they were absent from more than two consecutive dietitian meetings or consumed ≥3 inappropriate meal/supplement servings a week for ≥2 consecutive weeks at a time.

A 2-day food record was utilized to verify compliance to each respective diet (HP-IF-LC; HP-IF; HH). Based on our experience, a representative sample of only 2 days was adequate to assess subjects' stable and consistent intake during each stage. If necessary, subjects were instructed to record food intake every day. Food records were filled out by the participants at weeks 0, 11, and 63. A registered dietitian and a research team member provided instructions to the participants on making detailed records of portion sizes and food items. The dietary information was subsequently recorded into the food analysis program, The Food Processor SQL Edition (version 10.2.0 ESHA Research, Sale, OR, 2012). Single trained operators (E.W.) performed the analysis in order to eliminate inter-investigator variation. Each participant was also given a checklist in an effort to help adhere to the IF day regimen.

### Laboratory testing procedures

#### BW and physical activities assessment

Body weight was measured using an electronic scale during each testing visit without shoes and in minimal clothing. Standard BMI measurements were obtained by dividing the participant's weight (kg) by the square of their height (m^2^). Free-living, daily activity was monitored by an Actical accelerometer secured on the waist to ensure consistent levels of activity between the participants (Esliger et al., 2007; Hooker et al., 2011).

#### Blood lipid determination

A 12-h fasted venous blood sample (~20 ml) was obtained at baseline (week 0) and post-intervention (weeks 12 and 64). Blood was collected into EDTA-coated vacutainer tubes and centrifuged (Hettich Rotina 46R5) for 15 min at 2500 rpm at 4°C. After separation, plasma was stored at −70°C in small aliquots until analyzed. TC, high-density lipoprotein (HDL) cholesterol, and TG were assessed using the Cholestech LDX blood analysis system (Hayward, CA). Test-retest intraclass correlation (*r*) and coefficient of variation (CV) with *n* = 15 is: *r* = 0.95, CV = 3.2%, and *r* = 0.97, CV = 5.3%, for TC and HDL cholesterol, respectively.

#### Vascular compliance measurement

Vascular compliance can be measured non-invasively and gives information regarding cardiovascular disease risk, even in healthy individuals (Ring et al., 2014). Resting heart rate (HR) and systolic and diastolic BP (SBP; DBP) were obtained in the supine position as previously described (4, 6, 7). HR and BP were obtained by the same investigator (E.W.) following a minimum of 10 min of quiet resting.

The Arteriograph (version 1.10.0.1, TensioMed Kft., Budapest, Hungary) device uses an upper arm cuff inflated to >35 mmHg above the subjects' actual systolic pressure. This causes a small diaphragm in the brachial artery to develop along the upper border of the over-pressurized cuff. A pulse wave is created as the central pressure changes, forming an early (direct) systolic wave (P_1_), late (reflected) systolic wave (P_2_), and diastolic wave(s) (P_3_). The device is able to record each of these suprasystolic pressure changes.

Initially, the Arteriograph measures the systolic and diastolic BP oscillometrically, and then decompresses the cuff. Within a few seconds, the device re-inflates the cuff, first to the actual measured diastolic BP followed by the suprasystolic pressure, which is 35 mmHg over the actual systolic BP. The device records the signals from both pressure levels for 8 s. A computer receives all of the signals sent wirelessly by the device. Using the software, the augmentation index is determined by the following formula:

$$\begin{array}{l} \text{Aix}\left(\%\right)=\left({\text{P}}_{2}-{\text{P}}_{1}\right)/\text{PP}\times \text{100} \end{array}$$

where P_1_ reflects the early direct wave's amplitude; P_2_ refers to the late reflected systolic wave's amplitude; and PP equals the pulse pressure. Augmentation index was calculated for the brachial artery (brachial augmentation index; BAIX) and for the aorta (central augmentation index; CAIX). CAIX values are produced by the Arteriograph based on the correlation between previous simultaneously measured brachial and aortic augmentation indices. The aortic pulse wave velocity (PWVao) is determined by the wave reflection generated from the early direct pulse wave as it is reflected back primarily from the aortic bifurcation. Return time (RT) is determined by measuring the time interval between peaks from the early direct (P_1_) and reflected late (P_2_) systolic waves. The PWVao calculations were measured using the distance from the upper edge of the pubic bone to the sternal notch (Jugulum-Symphisis¼), as this provides the closest approximation of the true aortic length (Sugawara et al., 2008; Horváth et al., 2010). The parallel straight-line distance between these anatomical points was measured to allow for the calculation of the PWVao with the following formula:

PWVao(m/s) = [Jug – Sy(m)]/[(RT/2(s)]

Finally, calculation of the blood pressure using the Arteriograph was based on algorithms that have been previously validated (Németh et al., 2002; Horváth et al., 2010).

### Statistical analysis

Statistical analysis was performed using SPSS software (Ver. 21; IBM-SPSS). All values are reported as means ± SE. Before the start of the study, sample size was determined through power analysis based on the major outcome variables (blood lipids and arterial compliance) to achieve an effect size of 0.25 with 80% power at alpha 0.05 based on the preliminary data. This analysis determined that *n* = 12 were required to detect significant differences between groups. A 2 × 2 factor repeated measures ANOVA (RMANOVA) was performed for the WL (HP-IF-LC, weeks 0–12) phase (sex; M/F and time; control baseline vs. 12 Weeks) and the WL-M (weeks 13–64) phase (group; HP-IF/HH and time; 13 weeks vs. 64 weeks) to determine main effects as shown in the results. Bonferroni's method was performed if there was an interaction between variables. A multivariate ANOVA was also performed as an additional analysis. Pearson's correlation coefficients were used to assess the relationships between body fat, blood lipids, cardiovascular function, and arterial compliance during WL and WL-M phases. The significance was set at *p* < 0.05, and trends were noted for 0.05 < *p* < 0.1. Percent change for dependent variables was calculated as $\frac{\left(measurement\;2\right)\;-\;\left(measurement\;1\right)}{measurement\;1}*100$.

## Results

### WL (Phase 1, HP-IF-LC diet)

**Figure 2:**
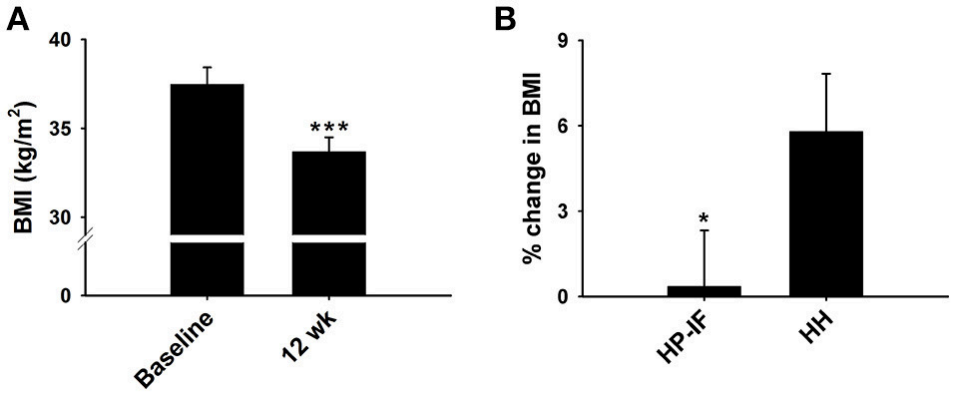
**Dietary effects on BMI during phase 1 (A) and phase 2 (B) collapsed across genders**. **(A)** Effect of 12 week HP-IF-LC intervention (Phase 1) on BMI (*n* = 40). **(B)** Percent change in BMI between HP-IF and HH groups during Phase 2 (*n* = 24). ^***^Significant difference compared to baseline (*p* < 0.001). ^*^Trend for significant difference compared to HH group (*p* = 0.069). BMI, body mass index; HP-IF, high-protein, intermittent fasting; HH, heart healthy.

Forty participants completed Phase 1 of this study. Descriptive baseline characteristics of the participants are shown in Table 3. Results of the repeated-measures ANOVAs for our dependent variables are displayed in Table 4. No gender-by-time interactions were found for any variables after Phase 1. The average loss of BW reached 10% of original BW following the 12-week WL (10.4 ± 0.6%, *p* < 0.001). BMI was also significantly decreased post Phase 1 compared to baseline (BMI: 37.5 ± 0.9 vs. 33.7 ± 0.8 kg/m^2^, *p* < 0.001; Figures 2A, **4A**). However, there was no difference observed in BMI at week 12 between those who chose to enter either the HH or HP-IF groups.

**Table 3:** **Baseline (week 0) characteristics of participants for WL (Phase 1)**.

	**Male (*n* = 21)**		**Female (*n* = 19)**			**Total (*n* = 40)**	
**Characteristics**	**Mean**	***SE***	**Mean**	***SE***	***p*-value**	**Mean**	***SE***
Age (years)	46.1	1.5	50.0	2.3	0.163	48.0	1.4
Weight (kg)	120.1	4.8	99.5	2.8	0.001	110.3	3.3
Height (cm)	179.0	1.7	163.1	1.0	0.000	171.4	1.6
BMI (kg/m^2^)	37.5	1.5	37.4	1.1	0.945	37.5	0.95
HR (bpm)	65.0	1.9	64.7	2.8	0.430	64.9	1.6
SBP (mmHg)	127.7	2.1	121.9	2.7	0.034	125.2	1.7
DBP (mmHg)	81.7	2.4	76.9	2.6	0.098	79.5	1.8

*BMI, body mass index; HR, heart rate; SBP, systolic blood pressure; DBP, diastolic blood pressure*.

**Table 4:** **Results of weight loss phase (Phase 1)**.

	**Gender**	**Baseline**	**12 weeks**	***p*-value (interaction)**	***p*-value (time)**	***p*-value (gender)**
BMI (kg/m^2^)	M	38.3 ± 1.6	34.3 ± 1.3	0.715	< 0.001^*^	0.598
	F	37.4 ± 1.1	33.7 ± 1.0			
TC (mg/dL)	M	188.2 ± 7.9	160.8 ± 6.6	0.670	< 0.001^*^	0.219
	F	199.6 ± 9.6	168.7 ± 8.2			
LDL (mg/dL)	M	114.6 ± 6.7	104.1 ± 6.4	0.505	0.036^*^	0.644
	F	121.0 ± 6.9	104.0 ± 5.8			
HDL (mg/dL)	M	48.2 ± 3.1	43.5 ± 2.8	0.846	0.006^*^	0.550
	F	51.1 ± 3.1	45.7 ± 2.7			
TG (mg/dL)	M	134.2 ± 17.7	84.9 ± 10.7	0.686	< 0.001^*^	0.307
	F	144.3 ± 13.7	102.7 ± 9.1			
HR (bpm)	M	65.5 ± 2.2	60.8 ± 2.3	0.878	0.017^*^	0.867
	F	64.8 ± 2.8	60.6 ± 2.3			
SBP (mmHg)	M	126.3 ± 2.0	116.5 ± 2.5	0.426	0.002^*^	0.195
	F	121.9 ± 2.7	115.3 ± 2.0			
DBP (mmHg)	M	80.1 ± 1.8	72.6 ± 1.8	0.783	< 0.001	0.158
	F	76.9 ± 2.6	68.7 ± 1.8			
PWVao (m/s)	M	7.8 ± 0.4	7.2 ± 0.3	0.571	0.001^*^	0.764
	F	7.8 ± 0.3	6.9 ± 0.3			
BAIX (%)	M	−27.0 ± 6.5	−37.3 ± 4.4	0.289	0.134	0.035^**^
	F	−10.6 ± 7.4	−12.6 ± 7.3			
CAIX (%)	M	24.0 ± 3.3	18.8 ± 2.2	0.289	0.133	0.035^**^
	F	32.3 ± 3.7	31.2 ± 3.7			
Return Time (s)	M	142.4 ± 6.0	157.4 ± 4.9	0.752	< 0.001^*^	0.101
	F	128.3 ± 5.4	146.1 ± 5.1			

Data presented as mean ± SE. Return time refers to the time intervals between peaks from the early direct and reflected late systolic waves.

* Significant difference based on time (p < 0.05);

** *Significant difference between genders (p < 0.05). BMI, body mass index; TC, total cholesterol; LDL, low-density lipoprotein; HDL, high-density lipoprotein; TG, triglycerides; HR, heart rate; SBP, systolic blood pressure; DBP, diastolic blood pressure; PWVao, pulse wave velocity; BAIX, brachial augmentation index; CAIX, central augmentation index; M, male; F, female*.

**Figure 3:**
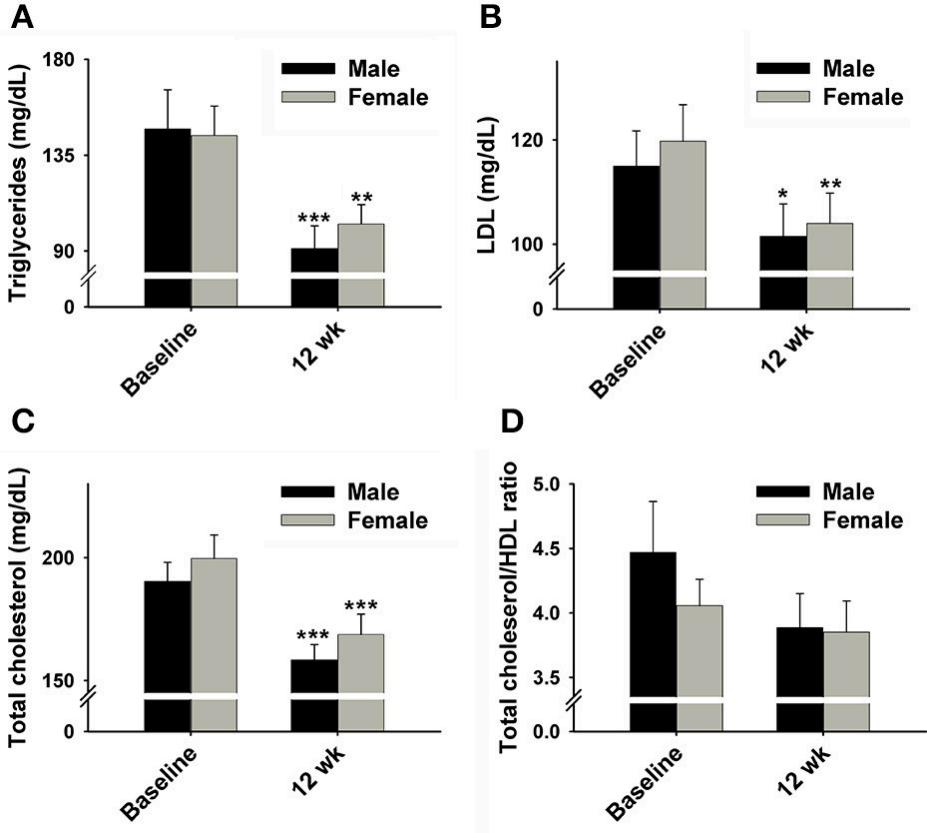
**Effect of 12 week HP-IF-LC intervention (Phase 1) on (A) triglycerides, (B) LDL, (C) total cholesterol, (D) total cholesterol/HDL ratio of males and females**. Significant difference compared to baseline of the same gender indicated by: ^*^p < 0.05; ^**^p < 0.01; ^***^p < 0.001. LDL, low-density-lipoprotein; HDL, high-density-lipoprotein.

**Figure 4:**
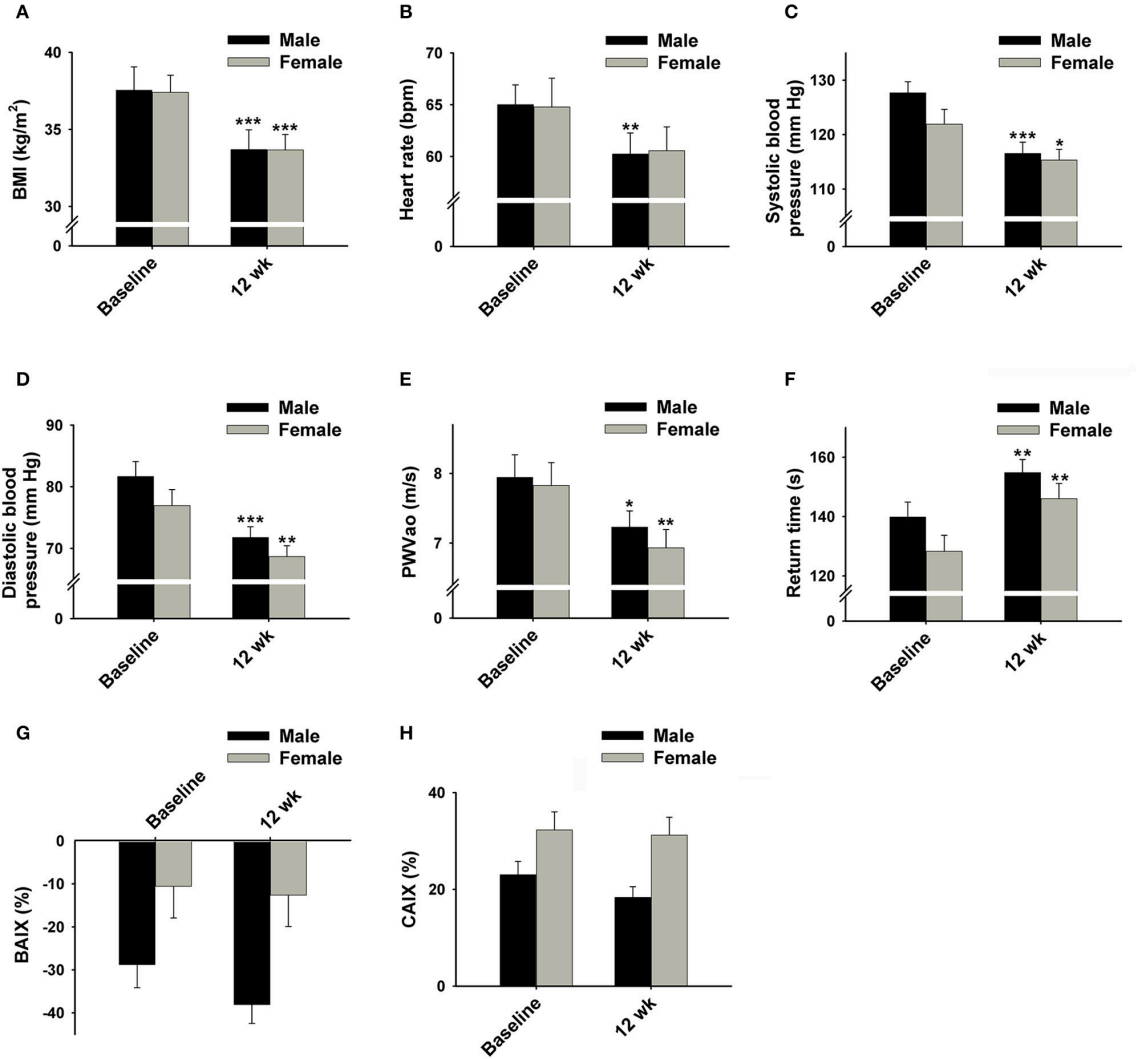
**Effect of 12 week HP-IF-LC intervention (Phase 1) on (A) BMI, (B) heart rate, (C) systolic blood pressure, (D) diastolic blood pressure, (E) PWVao, (F) return time (the time intervals between peaks from the early direct and reflected late systolic waves), (G) BAIX, and (H) CAIX of males and females**. Significant difference compared to baseline of the same gender indicated by: ^*^p < 0.05; ^**^p < 0.01; ^***^p < 0.001. BMI, body mass index; PWVao, aortic pulse wave velocity; BAIX, brachial augmentation index; CAIX, central augmentation index.

Significant decreases were also found in the levels of plasma TG, LDL, and TC following the WL (Figures 3A–D). As expected, HR and BP were also significantly decreased following the 12 weeks WL (Figures 4B–D); however, HR only decreased in male subjects.

Moreover, our results revealed that PWVao, an important measurement of arterial stiffness, was significantly decreased whereas the return time (RT) was increased following Phase 1 (Figures 4E,F). There were not significant changes in BAIX or CAIX (Figures 4G,H).

Percent changes in anthropometrics (BW and BMI) were significantly positively correlated with the corresponding changes in plasma TG (*r* = 0.30, 0.30, *p* < 0.05, respectively) and TC (*r* = 0.45, 0.48, *p* < 0.01, respectively), but not with the ratio of TC/HDL (Table 5). Interestingly, the percent change in CAIX was significantly correlated with HR, systolic BP, and diastolic BP (*r* = −0.29, *r* = −0.31, *r* = 0.29, *p* < 0.05, respectively).

**Table 5:** **Pearson correlation coefficients for the percent change in body weight/BMI and blood lipid profiles following the phase 1 study**.

	**TRG (% Δ)**		**LDL (% Δ)**		**TC (% Δ)**		**TC/HDL (% Δ)**	
**Body metrics**	***r*-value**	***p*-value**	***r*-value**	***p*-value**	***r*-value**	***p*-value**	***r*-value**	***p*-value**
Body weight (% Δ)	0.30	0.03^*^	0.22	0.09	0.45	0.00^**^	0.22	0.09
BMI (% Δ)	0.30	0.03^*^	0.27	0.05	0.48	0.00^**^	0.19	0.12

BMI, body mass index; TRG, triglycerides; LDL, low density lipoprotein; TC, total cholesterol; TC/HDL, total cholesterol/high density lipoprotein ratio.

* Significantly correlated (p < 0.05).

** *Significantly correlated (p < 0.01)*.

### WL-M (Phase 2, HP-IF, and HH diets)

Male and female participants were pooled for Phase 2. Twenty-four participants successfully completed Phase 2, while 16 were excluded due to drop-out and non-compliance. Descriptive characteristics of the participants are shown in Table 6, and the results of the repeated measures ANOVAs for our dependent variables are displayed in Table 7. TC, LDL, HDL, TG, HR, SBP, DBP, and return time increased over time in both groups. However, there were trends (0.05 < *p* < 0.1) for less regain in BMI (*p* = 0.069; Figure 2B) and LDL (*p* = 0.068) in the HP-IF group. There was also a reduced PWVao in the HP-IF group (*p* = 0.045) and increased CAIX over time in both groups (*p* = 0.084).

**Table 6:** **Baseline (week 12) characteristics of participants for WL-M (Phase 2)**.

	**HP-IF (*n* = 10)**		**HH (*n* = 14)**			**Total (*n* = 24)**	
**Characteristics**	**Mean**	***SE***	**Mean**	***SE***	***p*-value**	**Mean**	***SE***
Age (years)	50.9	3.1	50.0	1.8	0.791	50.4	1.6
Weight (kg)	90.4	2.4	95.1	3.7	0.306	93.1	2.4
Height (cm)	169.6	3.1	170.2	3.3	0.903	169.9	2.3
BMI (kg/m^2^)	31.6	0.82	33.1	1.3	0.076	32.5	0.81
HR (bpm)	57.4	2.8	58.7	2.7	0.607	58.2	1.9
SBP (mmHg)	114.0	3.3	110.9	2.5	0.534	112.2	2.0
DBP (mmHg)	68.9	2.6	69.3	2.0	0.859	69.1	1.5

*BMI, body mass index; HR, heart rate; SBP, systolic blood pressure; DBP, diastolic blood pressure*.

**Table 7:** **Results of weight loss maintenance phase (Phase 2)**.

	**Diet**	**Baseline (week 12)**	**64 weeks**	***p*-value (interaction)**	***p*-value (time)**	***p*-value (diet)**
BMI (kg/m^2^)	HP-IF	31.6 ± 0.8	31.8 ± 1.2	0.069^*^	0.102	0.264
	HH	32.2 ± 1.4	34.0 ± 1.4			
TC (mg/dL)	HP-IF	157.6 ± 8.4	184.4 ± 11.9	0.339	<0.001^**^	0.664
	HH	158.0 ± 12.8	195.2 ± 11.1			
LDL (mg/dL)	HP-IF	101.5 ± 7.7	113.1 ± 8.6	0.068^*^	0.002^**^	0.715
	HH	90.0 ± 8.5	116.8 ± 11.3			
HDL (mg/dL)	HP-IF	38.4 ± 3.2	50.1 ± 3.1	0.107	0.001^**^	0.365
	HH	46.8 ± 4.7	51.6 ± 4.5			
TG (mg/dL)	HP-IF	96.1 ± 12.0	124.1 ± 21.3	0.281	0.007^**^	0.764
	HH	91.0 ± 15.5	142.4 ± 22.9			
HR (bpm)	HP-IF	57.4 ± 2.8	60.0 ± 2.9	0.367	0.008^**^	0.677
	HH	57.5 ± 2.7	62.5 ± 1.7			
SBP (mmHg)	HP-IF	114.0 ± 3.3	130.8 ± 4.4	0.706	0.001^**^	1.000
	HH	112.8 ± 2.9	132.0 ± 5.3			
DBP (mmgHg)	HP-IF	68.9 ± 2.6	80.7 ± 2.9	0.987	<0.001^**^	0.514
	HH	70.8 ± 2.2	82.5 ± 3.8			
PWVao (m/s)	HP-IF	7.4 ± 0.6	7.0 ± 0.3	0.094^*^	0.665	0.992
	HH	6.9 ± 0.3	7.5 ± 0.4			
BAIX (%)	HP-IF	−9.2 ± 14.1	−10.2 ± 14.5	0.156	0.025	0.641
	HH	−36.8 ± 7.4	−15.9 ± 6.7			
CAIX (%)	HP-IF	30.9 ± 6.0	31.6 ± 5.8	0.288	0.084^*^	0.812
	HH	26.3 ± 3.7	32.7 ± 4.0			
Return Time (s)	HP-IF	146.6 ± 8.2	138.3 ± 6.8	0.220	0.002^**^	0.797
	HH	156.0 ± 7.4	134.7 ± 7.2			

Data presented as mean ± SE. Return time refers to the time intervals between peaks from the early direct and reflected late systolic waves.

* Trend for significant difference (0.05 < p < 0.1);

** *Significant difference based on time (p < 0.05). BMI, body mass index; TC, total cholesterol; LDL, low-density lipoprotein; HDL, high-density lipoprotein; TG, triglycerides; HR, heart rate; SBP, systolic blood pressure; PWVao, pulse wave velocity; BAIX, brachial augmentation index; CAIX, central augmentation index; HP-IF, high-protein intermittent fasting; HH, heart healthy*.

**Figure 5:**
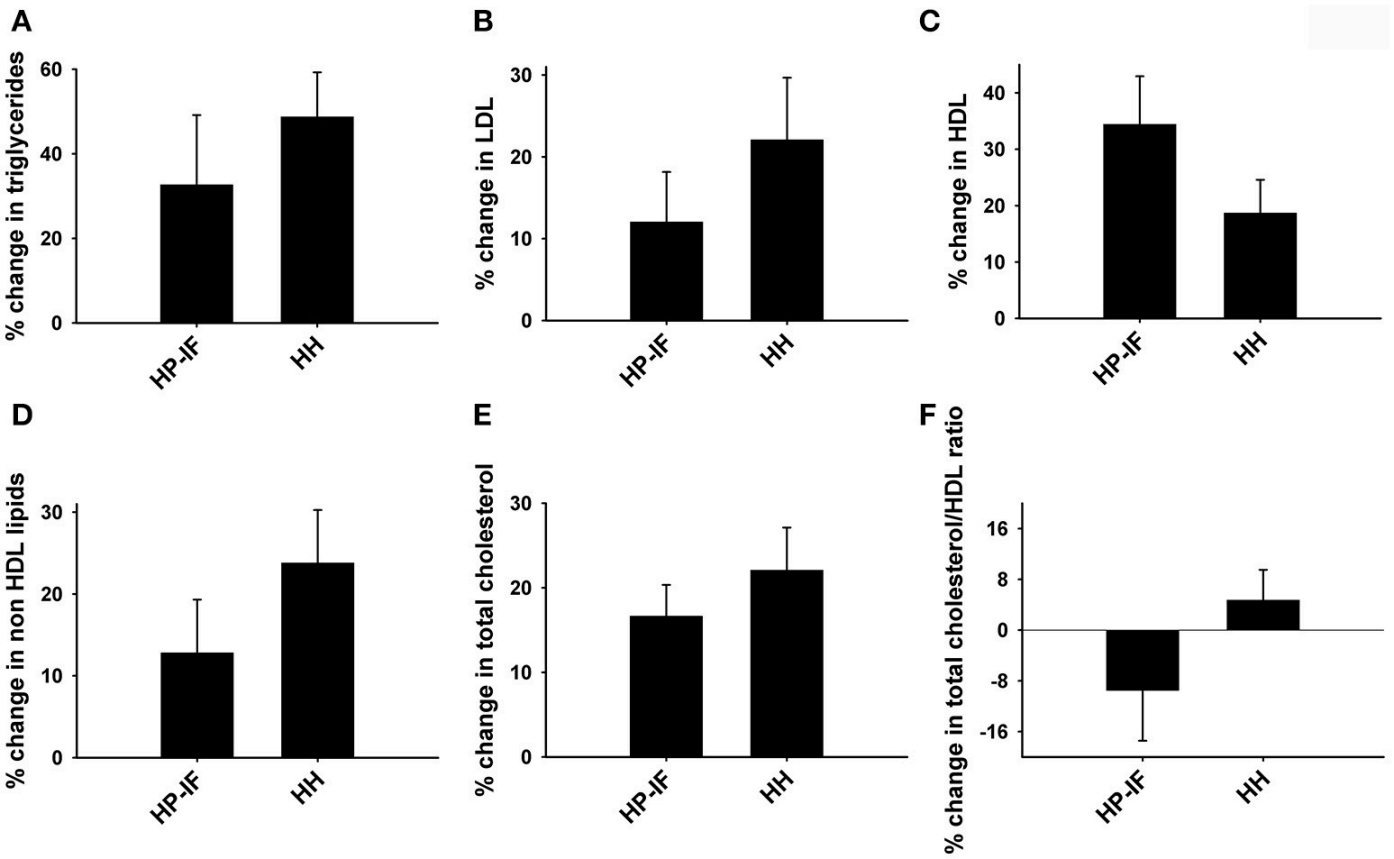
**Percent changes in (A) triglycerides, (B) LDL, (C) HDL, (D) non HDL lipids, (E) total cholesterol, and (F) total cholesterol/HDL ratio between the HP-IF and HH groups following Phase 2 (52 weeks)**. HP-IF, high-protein, intermittent fasting; HH, heart healthy; LDL, low-density lipoprotein; HDL, high-density-lipoprotein.

No significant difference was observed at the beginning of Phase 2 between the HP-IF and HH groups for any blood lipid components. Although there were no statistically significant differences in respect to the changes in blood lipid profiles between the HP-IF and HH groups following Phase 2, the HP-IF group demonstrated a tendency toward lower percentage increases compared to HH group in all lipid profiles except HDL (Figures 5A–F).

**Figure 6:**
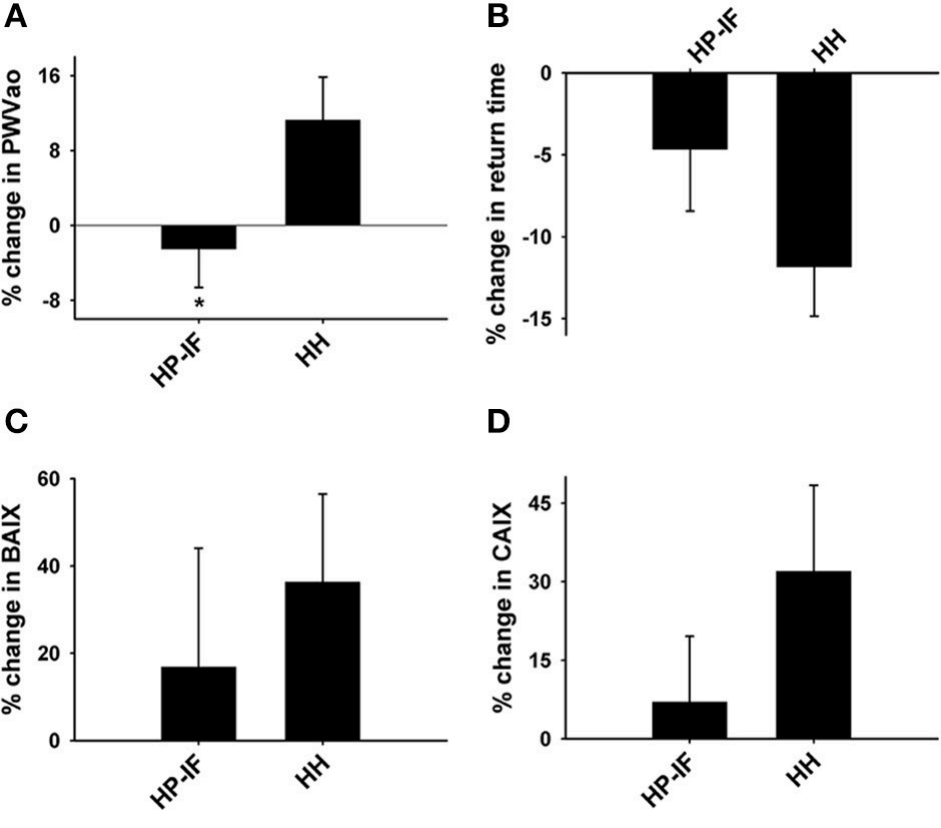
**Percent changes in (A) PWVao, (B) return time (the time intervals between peaks from the early direct and reflected late systolic waves), (C) BAIX, and (D) CAIX between the HP-IF and HH groups following Phase 2 (52 weeks)**. ^*^Significant difference from the HH group (*p* = 0.045), as assessed by repeated-measures ANOVA. PWVao, aortic pulse wave velocity; HP-IF, high-protein, intermittent fasting; HH, heart healthy; BAIX, brachial augmentation index; CAIX, central augmentation index.

Our results did not show a meaningful difference between the HP-IF and HH groups in terms of systolic BP, diastolic BP, or HR following phase 2. It is worth noting that the percent change in PWVao was much less in the HP-IF group than in the HH group following Phase 2 (−2.5 ± 4.1% vs. 11.2 ± 4.6%; Figure 6A). The difference in return time following the WL-M phase was also noticeable (HP-IF: −4.6 ± 3.8% vs. HH: −11.8 ± 3.0%, *p* = 0.220), although it was not statistically significant (Figure 6B). BAIX and CAIX percent changes are depicted in Figures 6C,D.

The percent change in systolic and diastolic BP was positively correlated with changes in BAIX (*r* = 0.52, *p* < 0.05; *r* = 0.63, *p* < 0.01, respectively). We also found that changes in TRG were positively correlated with changes in LDL, BAIX, and CAIX (*r* = 0.42, *r* = 0.49, *r* = 0.50, *p* < 0.05, respectively; Table 8).

**Table 8:** **Pearson correlation coefficients for the percent change in variables following the phase 2 study**.

	**LDL (% Δ)**		**BAIX (% Δ)**		**CAIX (% Δ)**		**PWVao (% Δ)**	
	***r*-value**	***p*-value**	***r*-value**	***p*-value**	***r*-value**	***p*-value**	***r*-value**	***p*-value**
Systolic BP (% Δ)	−0.22	0.17	0.52	0.01^*^	0.05	0.41	0.11	0.32
Diastolic BP (% Δ)	−0.19	0.20	0.63	0.00^**^	0.30	0.10	−0.28	0.12
TRG (% Δ)	0.42	0.03^*^	0.49	0.02^*^	0.50	0.01^*^	−0.27	0.12

LDL, low density lipoprotein; BAIX, brachial augmentation index; CAIX, central augmentation index; PWVao, aortic pulse wave velocity; TRG, triglycerides; BP, blood pressure.

* Significantly correlated (p < 0.05).

** *Significantly correlated (p < 0.01)*.

## Discussion

Previous studies have supported the independent effects of HP, HH, LC, or IF diets on WL success and cardiometabolic improvement (Arciero et al., 2006; Kelishadi et al., 2008; Clifton et al., 2009; Camhi et al., 2010; Klempel et al., 2012; Song et al., 2016). The current study provides convincing evidence that the combined HP-IF-LC diet successfully induces marked body WL, and is likely associated with reduced blood lipid levels and enhanced arterial compliance among men and women with obesity. During WL (Phase 1), the percent change in BW and BMI was significantly correlated with the changes in certain blood lipid variables such as TRG and TC. Subsequently in WL-M (Phase 2), subjects who adhered to the HP-IF diet experienced reduced weight regain and demonstrated better arterial compliance than those consuming the HH diet. Collectively, these data suggest that the HP-IF diet may be a new type of healthy diet which could be advantageous in maintaining the long-term health benefits from initial WL in overweight and obese individuals. Although significant WL occurred in both men and women, we found no sex effect in the parameters of our study.

The alarming rate of obesity in the United States is often attributed to the consumption of low-quality, high caloric diets (Bruemmer, 2012). Obesity was established as an independent risk contributor for CVD in the 26 plus-year follow-up from the original Framingham cohort study (Hubert et al., 1983). Numerous studies have investigated WL and the corresponding alterations in cardiovascular health and disease risk (Kelishadi et al., 2008; Clifton et al., 2009; Klempel et al., 2012; Mozaffarian et al., 2015; Pedersen et al., 2015). However, a major challenge we currently face is effectively maintaining successful WL and the accompanying health benefits. Therefore, it is paramount that researchers and clinicians develop WL strategies that are also safe and effective for aiding in long-term heart health (Bruemmer, 2012). While recidivism often appears to be unavoidable in long-term WL studies, maintaining even modest WL can be clinically significant (Stevens et al., 2001; Harsha and Bray, 2008).

Research has reported that short-term interventions similar to our HP-IF-LC diet effectively promote WL (Clifton et al., 2009; Klempel et al., 2012). One novel aspect of our study was comparing a traditional HH diet with a HP-IF diet using a 1-year follow-up to the initial 12-week WL period. We tracked changes in BMI, blood lipids, as well as cardiovascular and arterial compliance measures during both WL and WL-M phases. This could be attributed to the relatively higher protein content in the HP-IF diet, but the 1 to 2 days per month of IF likely exerted beneficial effects by counteracting normal weight gain. The individual and combined effects of HP and IF warrant further investigation.

The combined diet plan (HP-IF-LC) utilized in this study is not overly complex, and the modified IF that was included during the WL-M phase was only employed on 1 to 2 days per month. The HP-IF diet provided sufficient energy intake and was not intended for continued weight loss during the WL-M phase. Rather, it was included simply to more closely mimic the diet during the WL phase without the intended WL. Moreover, both HP and IF have been reported to induce significant health benefits, thus this was a logical dietary intervention to compare to the HH diet.

Previous research has shown that elevated resting HR can predict cardiovascular mortality in men and women (Fox et al., 2007; Cooney et al., 2010). Hypertension, which is associated with obesity, is also known to predispose individuals to CVD (Aucott et al., 2005). These serve as additional support for the necessity of long-term WL interventions which can sustain improvements in these significant cardiovascular variables. In addition, it is well-known that WL in hypertensive individuals is associated with a decrease in BP in short-term interventions either by caloric restriction, exercise, or both (Neter et al., 2003; Elmer et al., 2006; Harsha and Bray, 2008). Nevertheless, long-term studies on the effects of WL induced by different diets on BP are still lacking. As such, we measured systolic and diastolic BP, as well as resting heart rate, at weeks 0, 12, and 64. We observed a decline in systolic BP and diastolic BP following the 12-week WL Phase. However, the participants in the present study were, on average, categorized as having normal BP at study commencement, so the complete impact of these BP reductions is not entirely clear, although they could potentially aid in preventing a rise in BP over time. Some degree of weight relapse occurred during the WL-M phase, although body weight and BMI still remained below baseline levels. During the weight relapse, simultaneous increases in HR and BP were seen, but neither diet (HP-IF or HH) was shown to be more advantageous in lessening the recidivism in either BP or HR during the WL-M phase. In the future, extensive studies requiring a larger sample and longer-term interventions, possibly combined with exercise regimens, are needed to evaluate the advantages of the health-promoting effects of HP-IF on BP and HR changes.

The degree of WL has previously been linked to the reduction of CVD risk factors (Van Gaal et al., 2005). Consistent with previous studies (Melanson et al., 2003; Harder et al., 2004), we found that a 12-week WL program markedly reduced triglyceride levels and moderately lowered LDL (−25 and −11% in the present study), without improvements in HDL. In the future, moderate exercise combined with the HP-IF dietary intervention might be a promising approach to increase HDL levels while still reducing triglycerides and LDL. Following the 1 year WL-M phase, the improved lipid risk factors rebounded slightly, which was associated with recidivism of the weight gain. Although the weight relapse seems to be common for long-term WL studies, Wing et al. concluded that large or modest WL at 1 year is still clinically beneficial for diabetic patients with obesity (Wing et al., 2011), and it has been reported that acute weight loss affects long-term cardiovascular function (Corbi et al., 2002). Further studies should address the additional factors associated with CVD risk, such as the presence of diabetes and race difference, as well as the use of Framingham risk score to understand the beneficial effects of HP-IF diet.

An augmentation index is used as a measure of arterial stiffness, which has been demonstrated to adversely affect cardiovascular health (Nürnberger et al., 2002). For example, type 1 and type 2 diabetes are both characterized by increased arterial stiffness (Wilkinson et al., 2000). Our data revealed non-significant reductions in BAIX and CAIX after Phase 1, as well as a reduction in PWVao in the HP-IF group compared to the HH group in Phase 2. This finding suggests that the participants in the HP-IF group may have benefits from reduced arterial stiffness as a result of continuing the HP-IF diet for 1 year. Our data also suggest a positive relationship between the changes of BAIX, CAIX, and the change in TRG during phase 2. The beneficial outcome of the HP-IF diet may have clinical significance as studies have shown arterial stiffness to have a strong predictive value for cardiovascular events and all-cause mortality (Vlachopoulos et al., 2010). In accordance with our results, a study which examined patients with atherosclerotic disease found that the augmentation index was significantly correlated with both systolic BP and diastolic BP (Nürnberger et al., 2002). PWVao, another index of arterial stiffness, increases as a result of hypertension, atherosclerosis, or aging (Willum-Hansen et al., 2006). Research has shown that PWVao is a useful biomarker that can be successfully used to predict future cardiovascular risk in patients (Ben-Shlomo et al., 2014). Our findings are consistent with other studies showing that WL is associated with improved PWVao (Wildman et al., 2005; Barinas-Mitchell et al., 2006; Rider et al., 2010). Moreover, after the 1-year follow up, there is a lower PWVao over time in the HP-IF group as compared to the HH group, which relapsed to higher PWVao values.

Several significant strengths characterize the present study. First, there were two separate phases, WL and WL-M, with two different baselines which allowed for comparison between and within both interventions. Second, we diligently monitored the physical activity level and diet of all participants through both Phase 1 and Phase 2. On the other hand, the study also had some limitations. The age range of the cohort crosses over several reproductive stages of women (i.e., premenopausal, perimenopausal, and postmenopausal), which could affect the results. As is often the case in long-term human studies, the total number of our participants in phase 2 was less than that of phase 1 due to factors such as scheduling conflicts and non-compliance of subjects. Presumably, participants who finished the second phase were more motivated and committed to the study than those who dropped out. Therefore, we were unable to collect some follow-up data from drop outs, potentially increasing the possibility of false positive results. However, significant differences in baseline measurements did not exist between groups, meaning that the dropout and non-compliance did not produce systematic differences in the individuals in each group. The intensity of our dietary intervention may have been more stringent compared to other standard dietary interventions. As a result, future studies may need to be directed to a more practical protocol modified from current plans.

## Perspective and significance

In summary, a 12-week HP-IF-LC diet effectively improved BMI, lowered cholesterol, favorably altered cardiovascular variables, and reduced resting HR and BP. A novel finding is that a 1-year HP-IF intervention minimizes weight regain and improves BMI, leading to enhanced cardiovascular parameters. A comparison between this diet and other WL and WL-M trials should be performed in future studies, especially given that the HP-IF diet appears to be “advantageous” in maintaining the long-term health benefits from initial WL. A comparison WL group would be valuable in informing us whether the HP-IF-LC diet results in better metabolic and vascular outcomes than other traditional WL diets. Researchers should investigate the fasting glucose and HbA1C levels of the subjects, as diabetic and pre-diabetic subjects are prone to cardiovascular disease. In future studies, we will determine the exact effect of feedings during HP-IF-LC on gastrointestinal tract function, postprandial thermogenesis, RMR, nitrogen balance and glycosuria as compared to other diets. Furthermore, as previous studies have confirmed the involvement of reactive oxygen species in multiple CVD (Zuo et al., 2011; Zhu and Zuo, 2013), assessing the potential effects of different diets on the redox balance in the body may be an area of interest for future dietary studies at the molecular level. Further identification of an optimal role of cardiac preconditioning regimens and resistance training may be necessary as these methods may likely modulate lean body mass and muscle strength during WL (Zuo et al., 2013; Ataee et al., 2014).

## Author contributions

PA conception and design of research; PA performed experiments; LZ, GT, BP, EW, and PA analyzed data; LZ, FH, and PA interpreted results of experiments; LZ and BP prepared figures; LZ, FH, BP, and PA drafted manuscript; LZ, FH, BP, GT, and PA edited and revised manuscript; LZ, FH, GT, BP, EW, and PA approved final version of manuscript.

## Funding

This study was primarily supported (90%) by Isagenix International Grant #13-086 and (http://www.isagenix.com/?sc_lang=en-US) to PA. Additional support (10%) was provided by OSU-HRS Fund 013000.

### Conflict of interest statement

Isagenix International, a multilevel marketing company, funded this study to PA. However, the company did not have access to the data throughout the study, and no patents, products in development or marketed product is to declare. This does not alter our adherence to all the Frontiers in Physiology policies on sharing data and materials. All the other authors declare that the research was conducted in the absence of any commercial or financial relationships that could be construed as a potential conflict of interest.
